# Olfactory chemosensation extends lifespan through TGF-β signaling and UPR activation

**DOI:** 10.1038/s43587-023-00467-1

**Published:** 2023-07-27

**Authors:** Evandro A. De-Souza, Maximillian A. Thompson, Rebecca C. Taylor

**Affiliations:** 1 Neurobiology Division, Medical Research Council Laboratory of Molecular Biology, Cambridge, UK; 2 School of Biological Sciences, University of East Anglia, Norwich, UK

**Keywords:** Stress signalling, Gene regulation, Ageing

## Abstract

Animals rely on chemosensory cues to survive in pathogen-rich environments. In *Caenorhabditis elegans*, pathogenic bacteria trigger aversive behaviors through neuronal perception and activate molecular defenses throughout the animal. This suggests that neurons can coordinate the activation of organism-wide defensive responses upon pathogen perception. In this study, we found that exposure to volatile pathogen-associated compounds induces activation of the endoplasmic reticulum unfolded protein response (UPR^ER^) in peripheral tissues after *xbp-1* splicing in neurons. This odorant-induced UPR^ER^ activation is dependent upon DAF-7/transforming growth factor beta (TGF-β) signaling and leads to extended lifespan and enhanced clearance of toxic proteins. Notably, rescue of the DAF-1 TGF-β receptor in RIM/RIC interneurons is sufficient to significantly recover UPR^ER^ activation upon 1-undecene exposure. Our data suggest that the cell non-autonomous UPR^ER^ rewires organismal proteostasis in response to pathogen detection, pre-empting proteotoxic stress. Thus, chemosensation of particular odors may be a route to manipulation of stress responses and longevity.

## Abstract (web summary)

De-Souza, Thompson and Taylor demonstrate that pathogen-associated odorants can activate the UPR cell non-autonomously in *C. elegans* via neuronal TGF-β signaling, leading to extended lifespan and enhanced clearance of toxic proteins.

## Main

To adapt and survive, organisms must be able to detect and respond to environmental changes. In animals, this is mediated by the sensory nervous system, which activates defensive responses upon identification of hazards, such as reduced oxygen availability, temperature increase or food shortage^1^. In addition, the detection of stress within cells activates cellular stress responses, such as the unfolded protein response of the endoplasmic reticulum (UPR^ER^), which respond to homeostatic imbalance by activating mechanisms that restore homeostasis^2^. As animals age, they lose this ability to recognize and respond to stress, resulting in increased mortality and age-related disease^1,3–5^. In particular, reduced activity of the IRE-1/XBP-1 signaling branch of the UPR^ER^ has been linked to brain aging and neurodegeneration, whereas genetic activation of XBP-1 can protect animals against proteotoxic insults^5,6^.

Recent evidence suggests that neurons can trigger the cell non-autonomous activation of cellular stress responses in peripheral tissues, leading to coordinated increases in organismal resilience and lifespan. Consistent with this, genetic activation of the UPR^ER^ in a subset of neuronal or glial cells can extend lifespan in *Caenorhabditis* *elegans* via neuronal signaling mechanisms that result in UPR^ER^ activation in distal tissues^7,8^. However, whether specific environmental situations or exogenous molecules can trigger the activation of the cell non-autonomous UPR^ER^ in wild-type animals remains unknown. We therefore decided to identify physiologically relevant cues that drive cell non-autonomous UPR^ER^ activation in *C. elegans*.

## Results

### Pathogen-associated odorants can activate the UPR^ER^

**Fig. 1:**
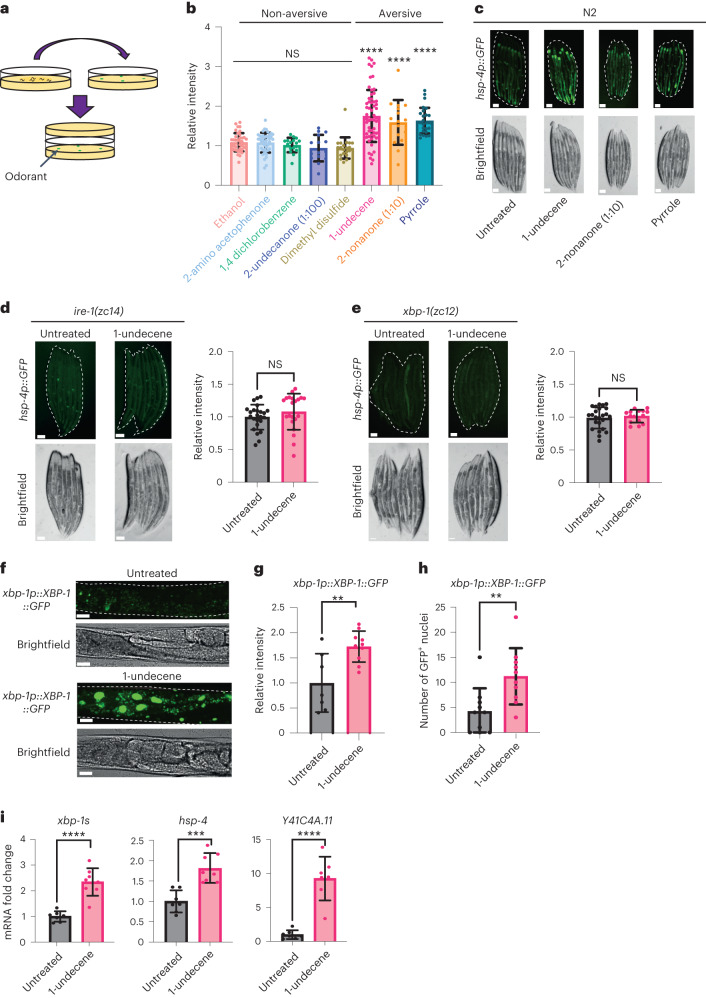
Pathogen-associated odor activates the IRE-1/XBP-1 branch of the UPR^ER^. **a**, Schematic showing the experimental setup for the odorant exposure assay. In brief, young adult worms were sealed for 12 h in NGM plates together with another NGM plate containing four spots of 3 μl of odorant. **b**, Fluorescence intensity of *hsp-4p::GFP* after odorant exposure. Quantification of *hsp-4p::GFP* expression was performed in ImageJ, and data were normalized to untreated *hsp-4p::GFP* animals. This assay was independently performed three times (*n* = 39, 42, 17, 15, 21, 60, 16 and 27 animals). Graphs show mean ± s.d. ****P < 0.0001 (one-way ANOVA with Dunnett’s multiple comparison test). **c**, Representative fluorescence microscopy images of worms untreated or exposed to 1-undecene, 2-nonanone (diluted 10×) and pyrrole for 12 h. This assay was independently performed three times. Scale bars, 200 μm. **d**,**e**, Representative fluorescence microscopy images and quantification of *hsp-4p::GFP* fluorescence in *ire-1(zc14)* (**d**) and *xbp-1(zc12)* (**e**) worms with or without exposure to 1-undecene odor for 12 h. These experiments were repeated four times (*n* = 26 and 21 animals for **d** and *n* = 25 and 16 animals for **e**). Scale bars, 200 μm. Graphs show mean ± s.d. NS, not significant (two-tailed unpaired Student’s *t*-test). **f**, Representative image. **g**, Quantification of fluorescence. **h**, Number of GFP^+^ nuclei in the intestine of worms expressing an *xbp-1p::xbp-1::GFP* transgene with or without exposure to 1-undecene for 8 h. This experiment was repeated three times (*n* = 7 and 10 animals for **g** and *n* = 10 and 10 animals for **h**). Scale bars, 200 μm. Graphs show mean ± s.d. ****P < 0.0001 and **P < 0.01 (two-tailed unpaired Student’s *t*-test). **i**, mRNA levels of *xbp-1s*, *hsp-4* and *Y41C4A.11* were measured by qRT–PCR in animals exposed to 1-undecene for 8 h relative to untreated worms (*n* = 7 and 8 biological replicates). Graphs show mean ± s.d. ****P < 0.0001 and ***P < 0.001 (two-tailed unpaired Student’s *t*-test). Precise *P* values are provided in Source Data. Source data

**Extended Data Fig. 1:**
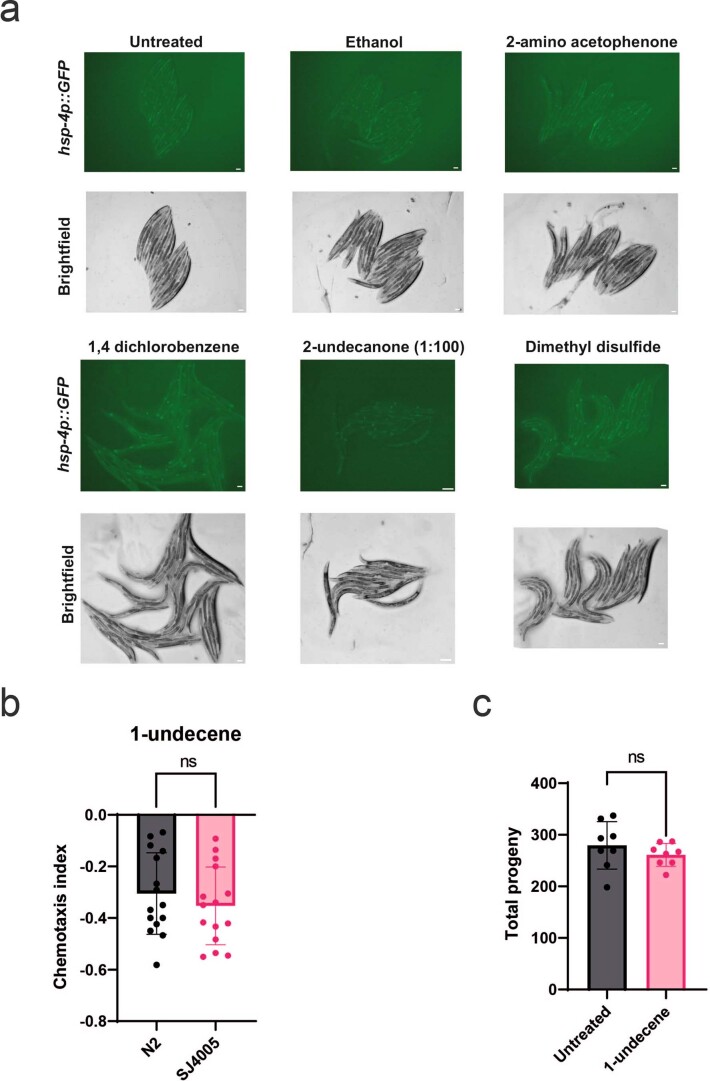
Non-aversive odorants do not induce UPR^ER^ activation and 1-undecene does not affect brood size. **a**, Representative images from the *hsp-4p::GFP* odorant screen. Experiments were repeated three times with at least 10 worms per group. Scale bars, 200 μm. **b**, Chemotaxis index (CI) of N2 and SJ4005 (*zcIs4* [*hsp-4p::GFP*]) worms exposed to 1-undecene. N = 15 biological repeats per group, 60 animals per replicate. Graph represents mean CI ± s.d. Ns, not significant (two-tailed unpaired Student’s t test). **c**, Number of eggs laid per worm in animals exposed to 1-undecene odor for 12 hours at day 1 of adulthood. n = 8 worms per group. Graphs show mean ± s.d. Ns, not significant (two-tailed unpaired Student’s t test). Source Data contains precise *P* values. Source data

Olfactory perception of bacteria alters gene expression in invertebrates^9^, and the immune response to *Pseudomonas spp* is associated with UPR^ER^ activation in *C. elegans*^3,10^. The smell of pathogenic bacteria can also sensitize the heat shock response in worms^11^, suggesting a possible link between olfaction and proteostasis. We therefore asked whether pathogen-associated odor could activate the cell non-autonomous UPR^ER^ in *C. elegans*. We exposed animals to a variety of odorant molecules secreted by pathogenic bacteria, including *Pseudomonas aeruginosa* and *Staphylococcus aureus*^12^, and monitored the expression of *hsp-4p::GFP*, a transcriptional reporter of UPR^ER^ activation. Notably, because the volatile molecules and the worms were placed on different plates, there was no direct contact between them (Fig. 1a). We observed that the UPR^ER^ could be activated in the intestine by exposure to three odorant molecules: 1-undecene, pyrrole and 2-nonanone (Fig. 1b,c and Extended Data Fig. 1a). Curiously, all three compounds have previously been linked to aversive behavioral responses in worms^13,14^ (Extended Data Fig. 1b). We decided to focus on 1-undecene in subsequent experiments. As previously observed by others^15^, exposure to higher concentrations of 2-nonanone caused lethal toxicity in a majority of the population; however, exposure to 1-undecene did not cause any overt alteration in the physiology of exposed animals, such as changes to brood size (Extended Data Fig. 1c).

**Extended Data Fig. 2:**
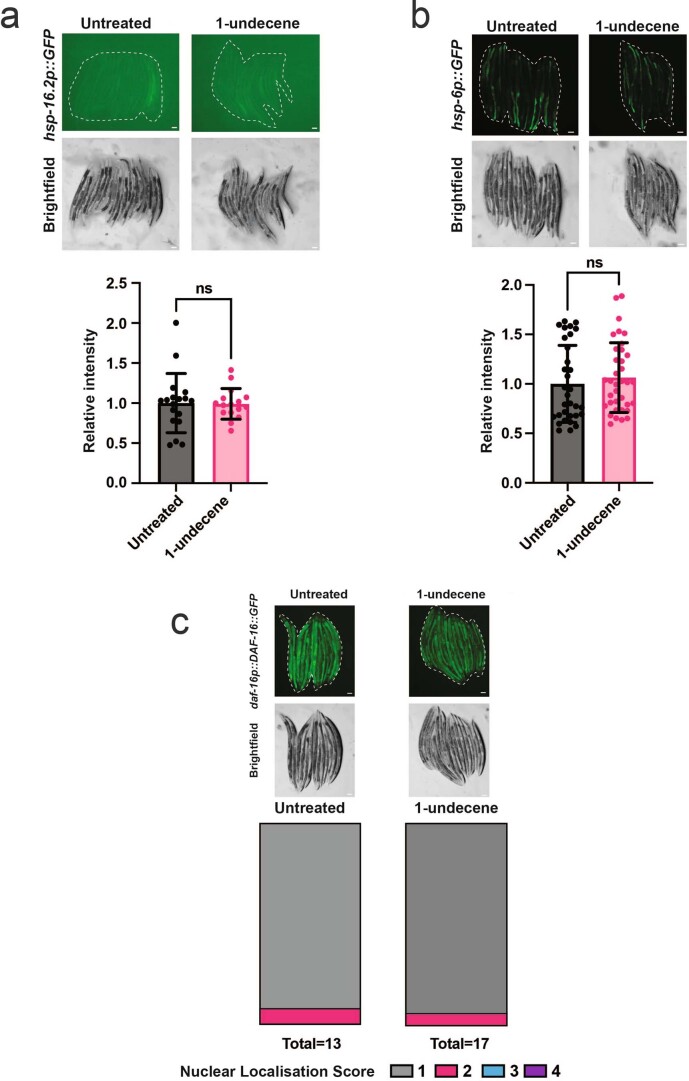
1-undecene exposure does not activate other stress response pathways. **a**,**b**, Representative fluorescence microscopy images and quantification of **a**, *hsp-16.2p::GFP* and **b**, *hsp-6p::GFP* fluorescence. These experiments were repeated three times. n = 18, 17 animals in **a** and n = 32, 36 animals in **b**. Scale bars, 200 μm. Graphs show mean ± s.d. Ns, not significant (two-tailed unpaired Student’s t test). **c**, Representative fluorescence microscopy images and quantification of the subcellular localization of DAF-16::GFP in worms expressing a *daf-16p::DAF-16::GFP* transgene. Worms were scored based on the number of intestinal cells that presented nuclear GFP localization, 1 = 0 cells (cytosolic GFP only), 2 = 2-4 cells, 3 = 5-8 cells, 4 = more than 8 cells. This experiment was repeated three times. n = 13, 17 animals. Scale bars, 200 μm. Source Data contains precise *P* values. Source data

**Extended Data Fig. 3:**
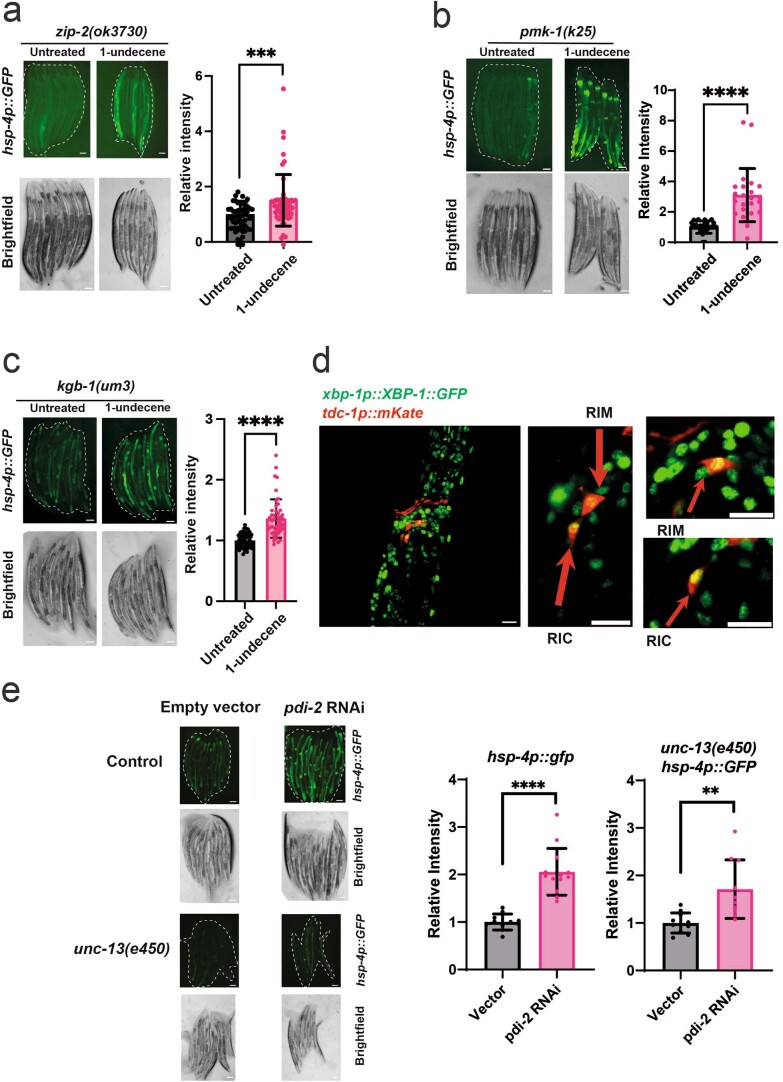
Activation of the UPR^ER^ in by 1-undecene odor does not require immune response regulators and occurs in RIM/RIC interneurons. **a, b, c**, Representative fluorescence microscopy images and quantification of *hsp-4p::GFP* fluorescence in **a**, *zip-2(ok3730)*, **b**, *pmk-1(k25)* and **c**, *kgb-1(um3)* animals with or without exposure to 1-undecene odor for 12 hours. Experiments were repeated three times. n = 50, 58 animals in **a**, n = 23, 18 animals in **b** and n = 54, 48 animals in **c**. Scale bars, 200 μm. Graphs show mean ± s.d. ***P < 0.001 in **a** and ****P < 0.0001 in **b** and **c** (two-tailed unpaired Student’s t test). **d**, Representative image of worms expressing a *tdc-1p::mKate; xbp-1p::xbp-1::GFP* transgene exposed to 8 hours of 1-undecene odor. Scale bars, 10 μm. Experiment repeated one time. Scale bars, 10 μm. **e**, Representative fluorescence microscopy images and quantification of *hsp-4p::GFP* fluorescence in *hsp-4p::GFP* animals with or without an *unc-13(e450)* mutant background grown from L1 larval stage on NGM plates containing bacteria harboring empty vector (L4440) or *pdi-2* RNAi. Data were normalized by samples treated with vector only. The experiment was repeated twice. n = 11, 13, 11, 10. Scale bars, 200 μm. Graphs show mean ± s.d. **P < 0.01, ****P < 0.0001 (two-tailed unpaired Student’s t test). Source Data contains precise *P* values. Source data

We found that mutation of the UPR^ER^ regulators *ire-1* or *xbp-1* abolished UPR^ER^ activation by 1-undecene odor, indicating that the IRE-1/XBP-1 signaling pathway is essential for activation of the UPR^ER^ by this compound (Fig. 1d,e). Consistent with this, an XBP-1s::GFP splicing reporter that expresses XBP-1s::GFP from an *xbp-1p::xbp-1::GFP* transgene only when *xbp-1* mRNA is spliced by IRE-1 (ref. ^8^) revealed an increase in XBP-1s::GFP within the intestinal cells of animals exposed to 1-undecene (Fig. 1f–h). Furthermore, we observed a significant increase in transcript levels of spliced *xbp-1* and two XBP-1s target genes (*hsp-4* and *Y41C4A.11*), confirming activation of the IRE-1/XBP-1 pathway by 1-undecene (Fig. 1i). Interestingly, we were unable to detect activation of other cellular stress response pathways, including nuclear DAF-16 localization and *hsp-16.2* (heat shock response) or *hsp-6* (mitochondrial UPR) upregulation, suggesting that the UPR^ER^ is specifically activated by pathogen-associated odor (Extended Data Fig. 2a–c). Finally, a recent study found that the *C. elegans* immune system can also be activated by olfactory perception of 1-undecene^16^. However, odor-induced UPR^ER^ activation is unlikely to be a downstream consequence of immune response activation, as animals with mutations in the key immunity transcription factor *zip-2*, or in the immunity-associated kinases *pmk-1* and *kgb-1*, still showed UPR^ER^ activation in response to 1-undecene (Extended Data Fig. 3a–c).

**Fig. 2:**
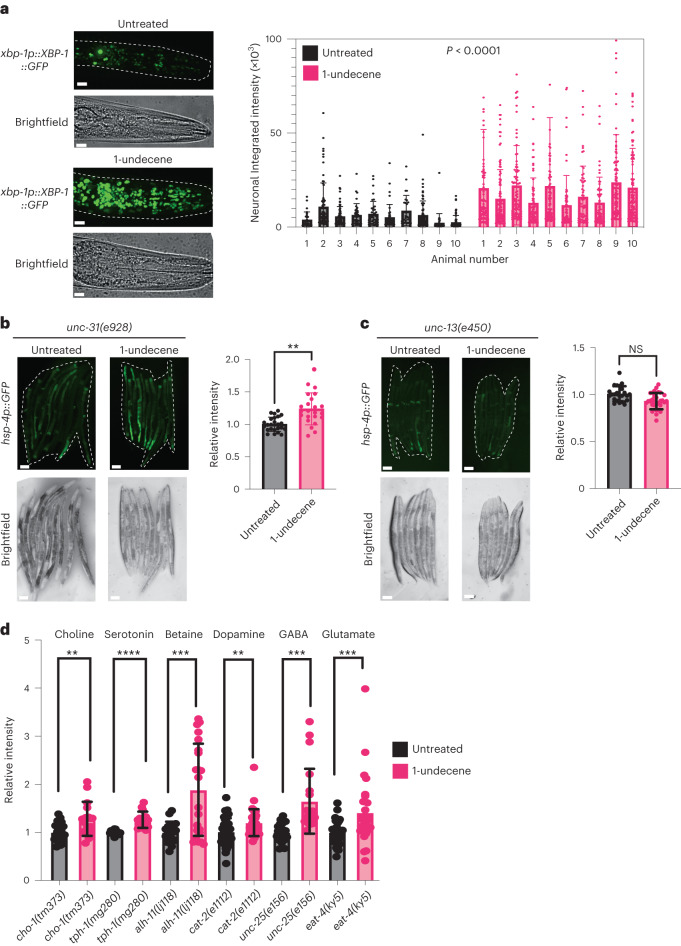
Neuronal signaling is required for downstream UPR^ER^ activation by 1-undecene exposure. **a**, Representative image and quantification of GFP^+^ cells in the head of worms expressing an *xbp-1p::xbp-1::GFP* transgene with or without exposure to 1-undecene for 8 h. This experiment was repeated three times with 10 worms per group. Scale bars, 10 μm. Graphs show mean ± s.d. Significance was calculated by unpaired Student’s *t*-test. **b**,**c**, Representative fluorescence microscopy images and quantification of *hsp-4p::GFP* fluorescence in *unc-31(e928)* (**b**) and *unc-13(e450)* (**c**) with or without exposure to 1-undecene odor for 12 h. These experiments were repeated three times (*n* = 24 and 23 animals for **b** and *n* = 23 and 30 animals for **c**). Scale bars, 200 μm. Graphs show mean ± s.d. NS, not significant and **P < 0.01 (two-tailed unpaired Student’s *t*-test) for **b** and **c**. **d**, Quantification of *hsp-4p::GFP* fluorescence in *cho-1*(*tm373)*, *tph-1*(*mg280*), *alh-1(ij118)*, *cat-2*(*e1112*), *unc-25(e156)* and *eat-4(ky5*) mutants. Intensity was normalized to untreated animals for each mutant strain. These experiments were repeated three times (*n* = 20, 16, 12, 15, 18, 22, 44, 39, 21, 20, 36 and 28). Graphs show mean ± s.d. **P < 0.01, ***P < 0.01 and ****P < 0.001 (two-tailed unpaired Student’s *t*-test comparison between untreated and 1-undecene). Precise *P* values are provided in Source Data. Source data

Previous work from our group and others demonstrated that neuronal signaling can activate the UPR^ER^ in peripheral tissues, such as the intestine^3,17^. We wondered whether signals produced by the nervous system were also responsible for odor-induced UPR^ER^ activation. We observed that animals exposed to pathogen-associated odor showed a significant increase in the number and fluorescence intensity of XBP-1s::GFP^+^ cells surrounding the pharynx (Fig. 2a), including neurons such as RIM and RIC (Extended Data Fig. 3d). To establish whether UPR^ER^ activation arising from 1-undecene exposure was cell non-autonomous in nature, we tested the dependency of this effect on the neuronal signaling regulators *unc-31* and *unc-13*—mutations in the former blocking release of neuropeptides from dense core vesicles and in the latter preventing the release of a range of signaling molecules, including small-molecule neurotransmitters^3,7^. We observed that the *hsp-4p::GFP* reporter was activated in the intestine of *unc-31(e928)* mutant animals (Fig. 2b), whereas the *unc-13(e450)* mutation entirely inhibited activation of the UPR^ER^ in the periphery (Fig. 2c), suggesting that a non-neuropeptide neuronal signal is involved in cell non-autonomous UPR^ER^ activation by exposure to 1-undecene. Notably, mutation of *unc-13* does not prevent animals from responding to cell-autonomous ER stress, as *hsp-4p::GFP* is still activated in animals exposed to RNA interference (RNAi) against *pdi-2* (Extended Data Fig. 3e)^18^.

### TGF-β signaling is required for odorant-induced UPR^ER^ activation

**Extended Data Fig. 4:**
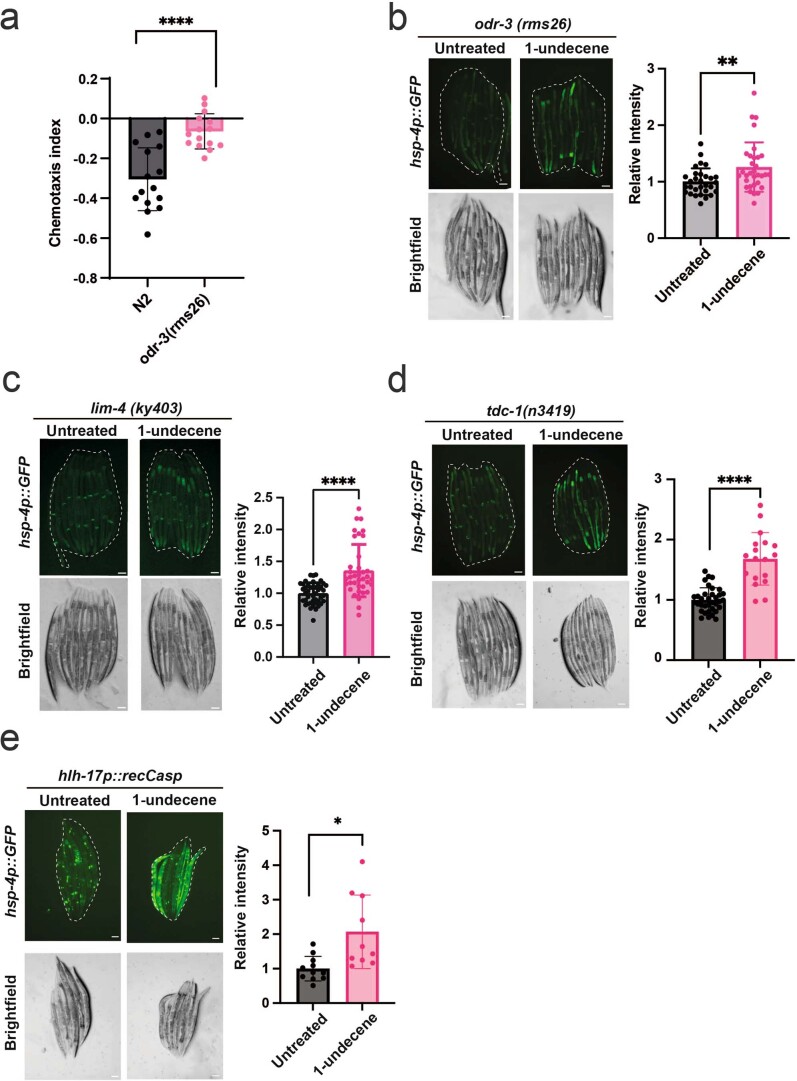
ODR-3, LIM-4, tyramine synthesis, and CEPsh glia are not required for UPR^ER^ activation by 1-undecene odor. **a**, Chemotaxis index of N2 and *odr-3(rms31)* animals following exposure to 1-undecene. n = 15 biological repeats per group, 60 animals per replicate (N2 data displayed is the same as Extended Data Fig. S1b as they were performed at the same time). Graphs show mean CI ± s.d. ****P < 0.0001 (two-tailed unpaired Student’s t test). **b, c, d, e** Representative fluorescence microscopy images and quantification of *hsp-4p::GFP* fluorescence in animals with **b**, *odr-3(rms31)*, **c**, *lim-4(ky403)* **d**, *tdc-1(n3419)*, and **e**, *nsIs180[hlh-17p::recCaspase-3, unc-122p::GFP]* backgrounds with or without exposure to 1-undecene for 12 hours. Experiments were repeated three (**b**, **c, e**) or four (**d**) times. n = 30, 32 animals in **b**, n = 42, 37 animals in **c**, n = 32, 18 animals in **d**, n = 11, 10 animals in **e**. Scale bars, 200 μm. Graphs show mean ± s.d. *P < 0.05, **P < 0.01, ****P < 0.0001 (two-tailed unpaired Student’s t test). Source Data contains precise *P* values. Source data

**Extended Data Fig. 5:**
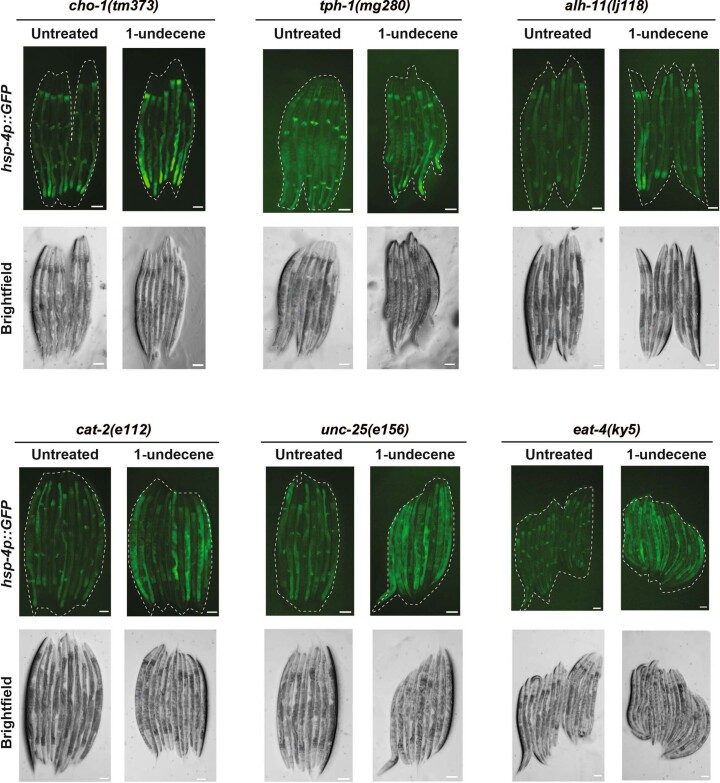
A range of neurotransmitters are dispensable for activation of the *hsp-4p::GFP* reporter transgene by 1-undecene exposure. Representative fluorescence microscopy images of *hsp-4p::GFP* fluorescence in *cho-1(tm373)*, *tph-1(mg280), alh-11(lj118), cat-2*(*e1112*), *unc-25(e156)*, and *eat-4(ky5)* backgrounds after exposure or no exposure to 1-undecene for 12 hours. Experiments were repeated three times with at least 8 worms per group. Scale bars, 200 μm.

The Gα protein ODR-3 was previously shown to be required for activation of the immune response by 1-undecene^16^. We therefore asked whether ODR-3 is also required for 1-undecene-induced UPR^ER^ activation. To do this, we used CRISPR to generate an *odr-3* deletion mutation in the *hsp-4p::GFP* background and confirmed that this mutation abolished the aversive behavioral response to 1-undecene (Extended Data Fig. 4a). However, we observed a full *hsp-4p::GFP* response in this *odr-3* null background, suggesting that this gene is not required for UPR^ER^ activation by 1-undecene (Extended Data Fig. 4b). The immune response to 1-undecene also requires the AWB sensory neurons^16^. However, a *lim-4* mutation, which results in dysfunction of AWB neurons, also failed to abolish 1-undecene-induced UPR^ER^ activation (Extended Data Fig. 4c). This suggests that the neuronal circuitry involved in the immune and UPR^ER^ activation responses to 1-undecene are different. In addition, tyramine synthesis is necessary for cell non-autonomous UPR^ER^ activation in strains constitutively expressing neuronal *xbp-1s*^8^. Unexpectedly, we found that *tdc-1*, a gene essential for the synthesis of tyramine, was not required for activation of *hsp-4p::GFP* in strains exposed to 1-undecene (Extended Data Fig. 4d). We also ruled out the possibility that the CEPsh glia are involved in this response, as animals in which these cells were genetically ablated still displayed increased *hsp-4p::GFP* levels after 1-undecene exposure (Extended Data Fig. 4e)^7^. We then tested mutants that fail to synthesize a variety of neurotransmitters, including dopamine, serotonin, GABA, glutamate, choline and betaine, for their ability to activate the UPR^ER^ in response to 1-undecene exposure, but we did not identify a role for any of these molecules (Fig. 2d and Extended Data Fig. 5).

**Fig. 3:**
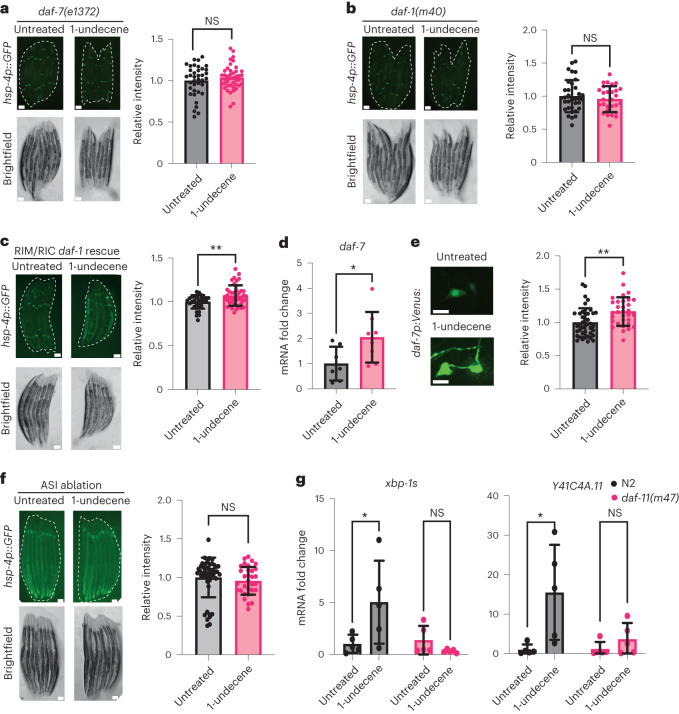
DAF-7/TGF-β signaling is required for odor-induced UPR^ER^ activation. **a**–**c**, Representative fluorescence microscopy images and quantification of *hsp-4p::GFP* fluorescence in *daf-7(e1372)* (**a**), *daf-1(m40)* (**b**) and *daf-1(m40)*;*ftEx205[tdc-1p::daf-1:gfp*] (**c**) strains with or without exposure to 1-undecene for 12 h. Each experiment was repeated four times (*n* = 38 and 42 animals in **a**, *n* = 34 and 28 animals in **b** and *n* = 34 and 42 animals in **c**). Scale bars, 200 μm. Graphs show mean ± s.d. NS, not significant (two-tailed unpaired Student’s *t*-test) or **P < 0.01 (two-tailed unpaired Student’s *t*-test with Welch’s correction). **d**, mRNA levels of *daf-7* were measured by qRT–PCR in animals exposed to 1-undecene for 8 h relative to untreated worms (*n* = 7 and 8 biological replicates). Graph shows mean ± s.d. *P < 0.05 (two-way ANOVA with Tukey’s multiple comparisons test). **e**, Representative fluorescence microscopy images and quantification of *daf-7p::Venus* fluorescence in ASI neurons after worms were exposed or not exposed to 1-undecene odor for 12 h. This experiment was repeated three times (*n* = 42 and 32 animals). Scale bars, 7 μm. Graph shows mean ± s.d. **P < 0.01 (two-tailed unpaired Student’s *t*-test). **f**, Representative fluorescence microscopy images and quantification of *hsp-4p::GFP* fluorescence in an ASI-ablated strain (*oyIs84[gpa-4p::TU#813 + gcy-27p::TU#814 + gcy-27p::GFP + unc-122p::DsRed]*). This experiment was repeated three times (*n* = 48 and 33 animals). Graph shows mean ± s.d. NS, not significant (two-tailed unpaired Student’s *t*-test). Scale bars, 200 μm **g**, mRNA levels of *xbp-1s* and *Y41C4A.11* were measured by qRT–PCR in animals exposed to 1-undecene for 8 h relative to untreated worms (*n* = 5 biological replicates). Graphs show mean ± s.d. NS, not significant and *P < 0.05 (two-way ANOVA with Tukey’s multiple comparison test). Precise *P* values are provided in Source Data. Source data

**Extended Data Fig. 6:**
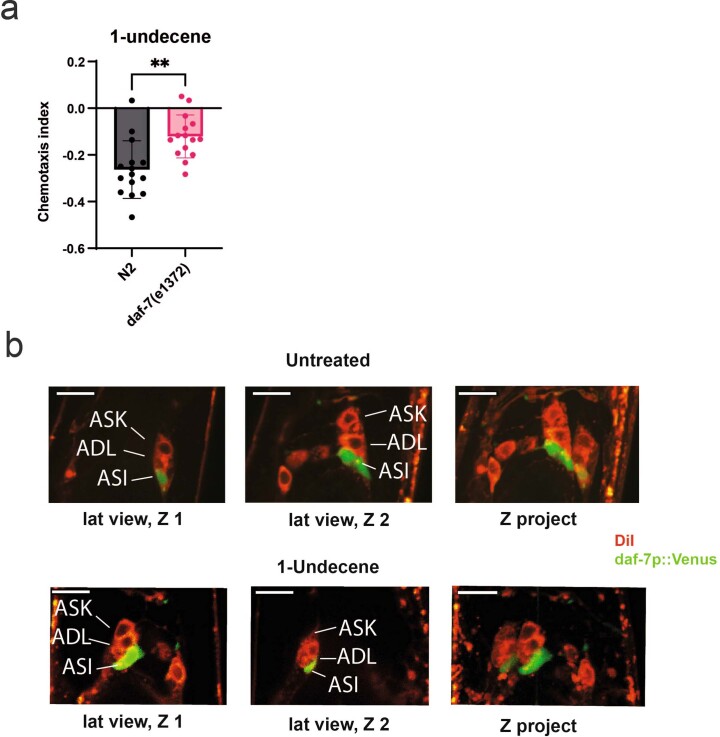
The *daf-7* pathway is activated by 1-undecene exposure and is involved in 1-undecene avoidance. **a**, Chemotaxis index of N2 and *daf-7(e1372)* strains exposed to 1-undecene. N = 15 biological repeats per group, 60 animals per replicate. Graphs show mean ± s.d. **P < 0.01 (two-tailed unpaired Student’s t test). Source Data contains precise *P* values. **b**, Confocal images of worms exposed to 1-undecene, expressing *daf-7p::Venus* and stained with DiI to label ciliated sensory neurons. The experiment was repeated 3 times. Scale bars, 20 μm. Source data

Worms avoid food containing pathogenic bacteria through aversive olfactory learning^19^. The same aversive behavior is seen in animals exposed to pathogen-associated molecules^20,21^. One signaling molecule that plays a key role in the neuronal circuits that govern these behaviors is transforming growth factor-beta (TGF-β)^20,22^. DAF-7, a worm homolog of TGF-β, is necessary for the avoidance of 2-nonanone^23^, a molecule whose odor induced UPR^ER^ activation in our initial odorant screen (Fig. 1b). We observed that DAF-7 is also necessary for behavioral avoidance of 1-undecene (Extended Data Fig. 6a). We therefore asked whether DAF-7/TGF-β is required for UPR^ER^ activation by 1-undecene. Strikingly, we found that *daf-7* was indeed necessary for UPR^ER^ activation after 1-undecene exposure (Fig. 3a). In addition, a mutation in a specific DAF-7 receptor, *daf-1*, completely inhibited 1-undecene-induced UPR^ER^ activation (Fig. 3b). Notably, DAF-1 is expressed in the RIM/RIC interneurons, and our previous work showed that UPR^ER^ activation in these neurons is sufficient to drive inter-tissue intestinal UPR^ER^ activation^8,20^. We found that rescue of DAF-1 in these interneurons alone was sufficient to partially restore UPR^ER^ activation in *daf-1(m40)* mutants (Fig. 3c). DAF-7 is primarily expressed in the ASI chemosensory neurons, and animals exposed to *P. aeruginosa* exhibit increased expression of *daf-7* (ref. ^20^). We therefore asked whether *daf-7* expression was elevated by chemosensation of 1-undecene. Indeed, *daf-7* mRNA levels were upregulated upon 1-undecene exposure (Fig. 3d). To confirm this, we also employed a *daf-7p::Venus* fluorescent reporter transgene and observed an increase in expression of *daf-7* only in the ASI neurons upon treatment with 1-undecene (Fig. 3e and Extended Data Fig. 6b). In addition, genetic ablation of the ASI neurons prevented UPR^ER^ activation by 1-undecene exposure (Fig. 3f). This suggests that an ASI-RIM/RIC neuronal circuit plays a role in the regulation of UPR^ER^ activation after odorant exposure.

Expression levels of *daf-7* have been linked to activation of the guanylate cyclase DAF-11 in ASI neurons during starvation^24^. We therefore asked whether DAF-11 is also required for UPR^ER^ activation upon 1-undecene exposure and observed that DAF-11 was indeed necessary for transcriptional upregulation of *xbp-1s* and its target gene *Y41C4A.11* (Fig. 3g). This suggests that DAF-11 is involved in the neuronal perception of 1-undecene odor and subsequent UPR^ER^ activation. Thus, our data implicate a TGF-β signaling circuit in connecting the recognition of pathogen-related odorants to inter-tissue regulation of the UPR^ER^.

### Chemosensory perception can extend lifespan and enhance proteostasis

**Fig. 4:**
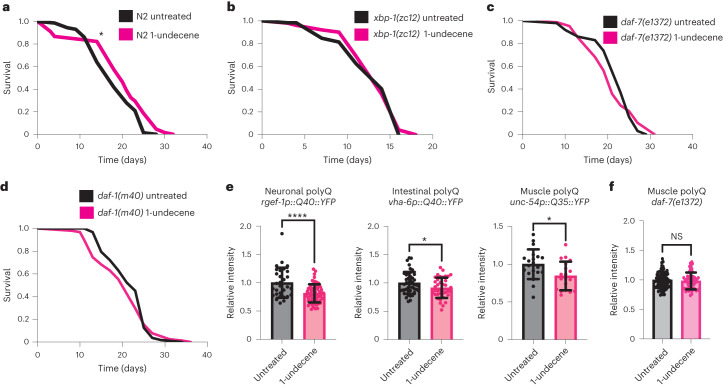
1-undecene odor increases *C. elegans* lifespan and reduces polyQ accumulation. **a**–**d**, Lifespan analysis of N2 wild-type (**a**), *xbp-1(zc12)* (**b**), *daf-7(e1372)* (**c**) and *daf-1(m40)* (**d**) animals with or without exposure to 1-undecene for 24 h at day 1 of adulthood. Graphs are plotted as Kaplan–Meier survival curves. *n* = 100–120 animals in each group in each of three biological replicates (**a**,**b**) and *n* = 50–100 in two biological replicates (**c**,**d**). *P < 0.05 (Mantel–Cox log-rank test). **e**, Animals expressing polyQ::YFP repeats in neurons, intestine or body wall muscle exposed to 1-undecene for 12 h at day 1 of adulthood and imaged 72 h after treatment. YFP levels were quantified using ImageJ and normalized to untreated animals. This experiment was repeated three times (*n* = 31 and 65 animals (neuronal); *n* = 21 and 20 animals (muscle); *n* = 44 and 39 animals (intestine)). Graphs show mean ± s.d. ****P < 0.0001 and *P < 0.05 (two-tailed unpaired Student’s *t*-test). **f**, *daf-7(e1372)* animals expressing polyQ::YFP repeats in body wall muscle exposed to 1-undecene for 12 h at day 1 of adulthood and imaged 72 h after treatment, as in **e**. This experiment was repeated three times with at least 15 worms per group. Graphs show mean ± s.d. NS, not significant (two-tailed unpaired Student’s *t*-test). Precise *P* values are provided in Source Data. Source data

**Extended Data Fig. 7:**
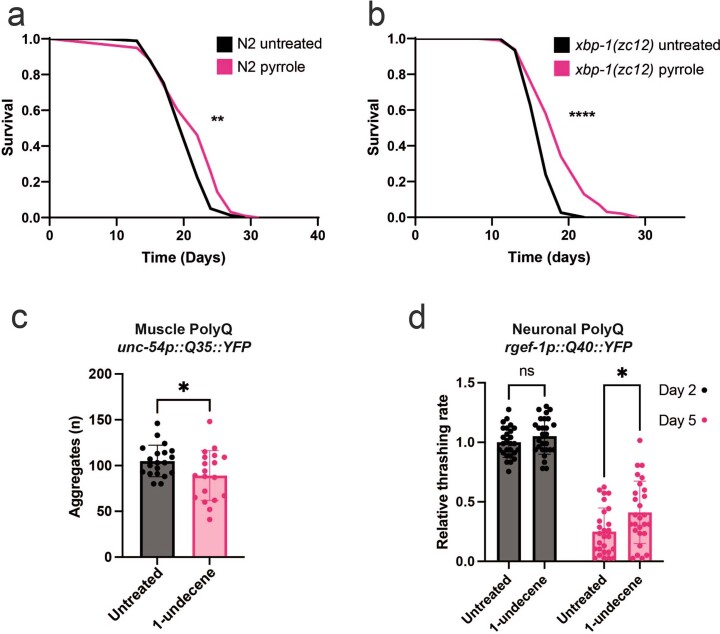
Pathogen-associated odor regulates longevity and proteostasis in *C. elegans*. **a, b**, Lifespan of **a**, N2 or **b**, *xbp-1(zc12)* strains exposed or not to pyrrole for 24 hours on day 1 of adulthood. Graphs are plotted as Kaplan-Meier survival curves. n = 90-100 animals in each group in 2-3 biological replicates. ****P < 0.0001, **P < 0.01 (Mantel-Cox log-rank test). **c**, The number of polyQ aggregates quantified in muscle cells on day 4 of adulthood in animals that were exposed or not for 12 hours to 1-undecene on day 1 of adulthood. n = 20, 19 worms per group in 3 biological replicates. Graphs show mean ± s.d. *P < 0.05 (two-tailed unpaired Student’s t test). **d**, Number of body bends quantified in worms expressing polyQ in neurons at day 2 or 5 of adulthood. Worms were exposed to 1-undecene or not for 16 hours on day 1 of adulthood. n = 30 worms per group in 3 biological replicates. Graphs show mean ± s.d. *P < 0.05, ns, not significant (two-tailed unpaired Student’s t test). Source Data contains precise *P* values. Source data

Activation of cellular stress responses is associated with increased lifespan and improved resistance to disease-associated toxic proteins^6,25,26^. This prompted us to ask whether 1-undecene exposure on the first day of adulthood could impact organismal lifespan and proteostasis. Excitingly, 1-undecene-exposed animals consistently had significantly longer lifespans than untreated animals (Fig. 4a and Supplementary Table 1). This increase in survival was dependent upon *xbp-1* (Fig. 4b and Supplementary Table 1), suggesting that 1-undecene odor extends lifespan through the activation of the UPR^ER^. Furthermore, 1-undecene-induced lifespan extension was also dependent upon *daf-7* and *daf-1*, confirming the importance of UPR^ER^ activation via TGF-β signaling downstream of 1-undecene exposure (Fig. 4c,d and Supplementary Table 1). Treatment with the UPR^ER^-inducing odorant pyrrole (Fig. 1b) also extended lifespan (Extended Data Fig. 7a and Supplementary Table 1). However, this extension of longevity was less consistent and was not dependent upon *xbp-1* (Extended Data Fig. 7b and Supplementary Table 1), suggesting the involvement of additional mechanisms.

**Extended Data Fig. 8:**
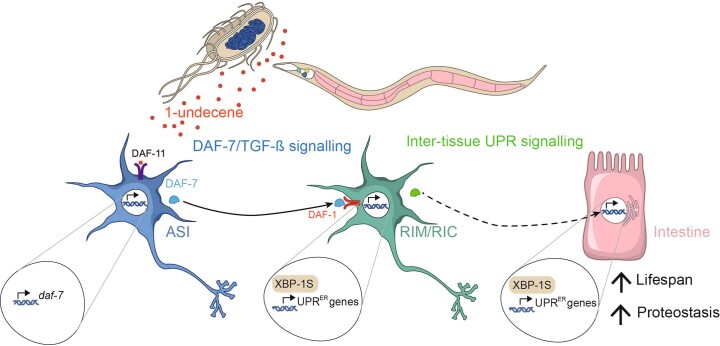
Model showing that olfactory stimulation, through exposure to 1-undecene, can activate the cell non-autonomous UPR^ER^ in *C. elegans* via TGF-β signaling.

To examine the impact of pathogen-related odor on a *C. elegans* model of neurodegeneration-associated proteotoxicity, we measured levels of YFP-tagged polyglutamine (polyQ) repeats in different tissues of the animal after 1-undecene exposure at day 1 of adulthood. Remarkably, 1-undecene induced a consistent decrease in levels of polyQ in all tissues examined (intestine, muscle and neurons) (Fig. 4e). It also decreased the number of polyQ aggregates observed in muscle cells (Extended Data Fig. 7c) and ameliorated motility decline in worms expressing neuronal polyQ (Extended Data Fig. 7d). This suggests that 1-undecene-induced UPR^ER^ activation enhances clearance of toxic proteins across the animal. Decreased polyQ levels upon 1-undecene exposure were also dependent upon *daf-7* (Fig. 4f). These results therefore suggest a model in which the neuronal perception of a pathogen-associated odorant molecule enhances organismal proteostasis and lifespan through TGF-β signaling and UPR^ER^ activation (Extended Data Fig. 8).

## Discussion

Previous studies reported the cell non-autonomous activation of the UPR^ER^ by signals from neurons and glia. In each case, however, transgenes driving *xbp-1s* were used to achieve this activation, and the evolutionary logic for the development of such systems has been unclear. Here we demonstrate that *C. elegans* can trigger a cell non-autonomous UPR^ER^ without such transgenes, in response to pathogen-associated odorant molecules that induce an aversive behavioral response. We reason that the cell non-autonomous UPR^ER^ may have evolved to enable animals to enhance defensive mechanisms in anticipation of the increased translation associated with an immune response or the direct proteostatic challenge of the pathogen itself. Animals that constitutively activate a PMK-1-driven immune response require *xbp-1* to survive the demands imposed by an active immune system^10^, suggesting that UPR^ER^ capacity is of critical importance in conditions that require an immune response.

Although the action of 1-undecene on *C. elegans* is likely a specific interaction informed by the complex evolutionary relationship between pathogen and host, existing evidence supports the idea that the broader principle underlying cell non-autonomous UPR^ER^ activation may be conserved. In mice, sensory perception of food activates pro-opiomelanocortin (POMC)-expressing neurons, resulting in hepatic *xbp-1* splicing as a predictive physiological response in anticipation of food consumption^27^. In addition, driving *xbp-1s* genetically in murine POMC neurons is sufficient to increase hepatic *xbp-1s* levels via a cell non-autonomous mechanism^17^. There are significant similarities between the roles of ASI neurons in the worm and the hypothalamus and POMC neurons in mice^28^. ASI neurons regulate food intake and food-seeking behavior through the action of DAF-7/TGF-β^29^. Similarly, POMC is expressed in subsets of cells, including neurons in the arcuate nucleus of the hypothalamus^30^, and POMC neurons also regulate food intake and energy expenditure via locomotion in some contexts^31^. Furthermore, expression of the TGF-β antagonist Smad7 in POMC neurons regulates peripheral glucose metabolism, suggesting that TGF-β signaling is also important for POMC neurons to achieve anticipatory, cell non-autonomous effects in the periphery^32^. These mammalian studies suggest that major interactions in the pathway we describe here are likely to be conserved in mammals.

Although earlier work showed that food-associated odor can prevent lifespan extension induced by caloric restriction^33,34^, the present study is, to our knowledge, the first demonstration that the perception of a specific odorant molecule can increase the lifespan of an animal. It was noted recently^35^ that a great many mechanisms that regulate aging in model organisms include cell non-autonomous protective pathways that are subject to neuronal control, often by sensory neurons. Dietary restriction-mediated longevity requires the UPR^ER^ (refs. ^36,37^) as well as functional ASI neurons expressing *daf-7* (refs. ^38,39^) and is regulated by olfactory perception^40^. Furthermore, cell non-autonomous regulation of the mitochondrial UPR^41^, heat shock response^42^, AMP-activated protein kinase (AMPK)^43^ and target of rapamycin complex 1 (TORC1)^44^, as well as lifespan regulation by temperature^45^ and the hypoxia response^46^, are all similarly orchestrated, with signals originating in sensory neurons leading via cell non-autonomous routes to regulation of pro-longevity pathways. Here we show that direct stimulation of chemosensory neurons can extend lifespan. We therefore speculate that directly manipulating the activity of sensory neurons via their sensory inputs and/or corresponding receptors may be a way to activate these pro-longevity pathways.

Finally, mounting evidence suggests that Ire1/Xbp1 activity is highly correlated with the pathophysiology observed in neurodegenerative disorders in animal models, including Alzheimer’s, Parkinson’s and Huntington’s diseases, and age-associated decline in the activation of this pathway may be associated with disease progression^47–49^. Activation of the UPR^ER^ through stimulation of sensory pathways by olfactory compounds may therefore represent a promising strategy to prevent the disease-related proteostasis collapse associated with aging.

## Methods

### *C. elegans* strains and maintenance

Strains were made in the course of this study, provided by the Caenorhabditis Genetics Center (CGC) or kindly gifted by other laboratories. A list of strains used in this work can be found in Supplementary Table 2. The CGC Bristol N2 hermaphrodite stock was used as wild-type. Worms were maintained at 20 °C on nematode growth medium (NGM) plates seeded with *Escherichia coli* OP50 using standard techniques^50^. For RNAi by feeding^51^, NGM plates were supplemented with 1 mM IPTG and 100 μg ml^−1^ carbenicillin and then seeded with HT115 bacteria harboring L4440 empty vector or the RNAi of interest. All RNAi used are from the Ahringer RNAi library (Source BioScience) and were confirmed by sequencing.

### Transgenic strain construction

The *odr-3(rms31)* mutant was generated by CRISPR using a dual crRNA *dpy-10* co-CRISPR strategy and a custom protocol based on previous methods^52,53^ and optimization for our laboratory. In brief, 1 µl of 320 µM solution of each CRISPR RNA (crRNA) and 0.5 µl of *dpy-10* crRNA (50 µM) was annealed to 0.4 µl of 100 µM trans-acting CRISPR RNA (tracrRNA) (Integrated DNA Technologies) by heating to 95 °C in a PCR machine and cooling to 4 °C at 0.1 °C s^−1^. Then, 0.5 µl of Cas9 protein (Invitrogen) was added, and the mixture was incubated for 10 min at 37 °C. Next, 0.5 µl of 100 µM stock of each repair template (target and *dpy-10*) was added, and the solution was made up to 20 µl with DPEC water. This mix was centrifuged for 30 min at 20,000*g* at 4 °C before injection. Oligonucleotides used in this study are provided in Supplementary Tables 3 and 4.

### Brood size measurement

Brood size was determined by counting the number of eggs laid per worm during their fertile period (from day 1 to day 4 of adulthood).

### Treatment with 1-undecene

To expose animals to the odor of 1-undecene (Sigma-Aldrich, 242527), worms were placed on NGM plates with a diameter of 60 mm, which were sealed with another 60 mm NGM plate on which was pipetted 4 × 3-μl drops of 1-undecene.

### Epifluorescence microscopy

To investigate the effect of 1-undecene on reporter transgene expression (for example, *hsp-4p::gfp*), worms were exposed to 1-undecene odor for 12 h in plates sealed with Parafilm M and then immobilized with 20 mM sodium azide (Sigma-Aldrich) and imaged using a Leica M205 FA microscope. To image worms expressing polyQ::YFP, animals were exposed to 1-undecene for 12 h on day 1 of adulthood and imaged on day 4 of adulthood. For DAF-16::GFP analysis, worms were scored based on the subcellular localization of GFP in intestinal cells, as described previously^24^. Worms were randomly selected from a synchronized population before imaging. Fluorescence values (mean intensity) were obtained by analyzing microscope images on ImageJ or Fiji.

### Confocal microscopy

Worms expressing *daf-7p::Venus* or *xbp-1p::xbp-1::GFP* transgenes were treated with 1-undecene for 8 h. They were then immobilized with 20 mM sodium azide (Sigma-Aldrich) and mounted on a 2% agarose pad. Animals were imaged on an LSM 710 confocal microscope using the ×40 and ×63 oil immersion objectives and on an Andor Revolution spinning disk microscope using the ×20 and ×60 water immersion objectives. All images were acquired using Leica LAS X (version 5.1.0) and analyzed using ImageJ (version 1.53e).

### RNA extraction and qRT–PCR

Approximately 300 young adult animals were collected with M9 after being exposed or not to 1-undecene for 8 h. TRIzol was added to samples, which were immediately frozen in liquid nitrogen. RNA isolation was carried out using the Direct-zol RNA MiniPrep Kit (Zymo Research) following the manufacturer’s instructions. RNA was quantified by NanoDrop. One microgram of RNA was used for cDNA synthesis with the QuantiTect Reverse Transcription Kit (Qiagen). Samples were diluted 2.5× after cDNA synthesis, and SYBR Select Master Mix (Applied Biosystems) was used for qRT–PCR on a Vii7 Real-Time PCR machine (Thermo Fisher Scientific) to quantify alterations in the transcript level of genes of interest. Data were analyzed using the comparative 2^−ΔΔCt^ method. A list of primers used in this work is provided in Supplementary Table 3.

### Chemotaxis assay

For odorant chemotaxis assays, chemotaxis was performed in a two-plate setup. On the lower plate, 1-undecene (test) or water (control) was placed approximately 2 cm from the center of the plate. Worms at day 1 of adulthood were placed on the center of the upper plate, and a test zone and a control zone were designated opposite the odorant, with the remaining space scored as the center zone^14^. Water mixed with sodium azide (1:1) was placed in the center of each the control and test zone. After 30 min, the number of worms in each zone was quantified, and the chemotaxis index was calculated by the formula: CI = (number of worms_test_ − number of worms_control_) / (total number of worms).

### Thrashing assay

Animals expressing neuronal polyQ were exposed to 1-undecene on day 1 of adulthood for 16 h. At day 2 or day 5 of adulthood, these animals were transferred to M9 solution, and the number of body bends per 30 s was quantified.

### Survival assays

Approximately 100 worms were exposed or not exposed to 1-undecene odor for 24 h at day 1 of adulthood. Worms were then placed on NGM plates containing 100 μg ml^−1^ FUDR and seeded with *E. coli* OP50 and were kept at 20 °C. Animals were monitored as alive or dead every second day by a blinded investigator, and data were analyzed on GraphPad Prism 8.4.2 software.

### Statistics and reproducibility

Statistical analysis was performed using GraphPad Prism 8.4.2 software. All bar graphs show the mean with error bars representing s.d. Appropriate tests for each experiment were chosen and are described (including tests for multiple comparisons) in the figure legends. With the exception of lifespan assays, data collection and analysis were not performed blinded to the conditions of the experiments. Unless specified otherwise in the figure legend, a minimum of three individual experiments were conducted for each assay. All replication efforts consistently yielded similar results. No animals were excluded from the analysis; however, for qRT–PCR experiments, samples that did not meet the predetermined quality control standards were excluded. Where used, *n* is immediately defined. Information regarding the number of repeats, number of animals per repeat and the results of the statistical tests performed are given in the figure legends. No statistical methods were used to pre-determine sample sizes, but our sample sizes are similar to those reported in previous publications from our group^6^. Data distribution was assumed to be normal, but this was not formally tested. Animals were randomly selected based upon developmental stage and not screened in any way before analysis.

### Reporting summary

Further information on research design is available in the Nature Portfolio Reporting Summary linked to this article.

## Supplementary information

Supplementary Tables 1–4.

Reporting Summary

## Data Availability

All data reported in this paper will be shared by the lead contact upon reasonable request. Any additional information required to re-analyze the data reported in this paper is available from the lead contact upon reasonable request. This paper does not report original code. Source data are provided with this paper.

## Source data

Source Data Fig. 1 Statistical Source Data.
Source Data Fig. 2 Statistical Source Data.
Source Data Fig. 3 Statistical Source Data.
Source Data Fig. 4 Statistical Source Data.
Source Data Extended Data Fig. 1 Statistical Source Data.
Source Data Extended Data Fig. 2 Statistical Source Data.
Source Data Extended Data Fig. 3 Statistical Source Data.
Source Data Extended Data Fig. 4 Statistical Source Data.
Source Data Extended Data Fig. 6 Statistical Source Data.
Source Data Extended Data Fig. 7 Statistical Source Data.
