# Supplementary material for: Olfactory chemosensation extends lifespan through TGF-β signaling and UPR activation
Source: Nat Aging. 2023 Jul 27;3(8):938–47. doi: 10.1038/s43587-023-00467-1 (PMC10432268; doi:10.1038/s43587-023-00467-1)
Supplement: Supplementary file 1 — Supplementary Tables 1–4. [file 43587_2023_467_MOESM1_ESM.pdf]

nature aging

Letter

https://doi.org/10.1038/s43587-023-00467-1

Olfactory chemosensation extends lifespan through TGF-β signaling and UPR activation

In the format provided by the authors and unedited

Table of Contents

1. **Supplementary Table 1. Survival information for lifespan experiments.**
2. **Supplementary Table 2. List of *C. elegans* strains used in this work.**
3. **Supplementary Table 3. List of qPCR primers used in this work.**
4. **Supplementary Table 4. List of CRISPR primers and oligonucleotides used in this work.**

Supplementary Table 1. Survival information for lifespan experiments.

Strain	Treatment	Median lifespan (days)	Control	Median lifespan (days)	*P* value (Mantel-Cox)
N2	+ 1-undecene	20	- 1-undecene	18	0.0227
N2	+ 1-undecene	20	- 1-undecene	18	0.0357
N2	+ 1-undecene	26	- 1-undecene	21	0.0185
N2	+ 1-undecene	25	- 1-undecene	21	0.0007
N2	+ 1-undecene	25	- 1-undecene	21	<0.0001
N2	+ 1-undecene	23	- 1-undecene	19	<0.0001
xbp-1(zc12)	+ 1-undecene	14	- 1-undecene	14	0.7015
xbp-1(zc12)	+ 1-undecene	18	- 1-undecene	20	<0.0001
xbp-1(zc12)	+ 1-undecene	22	- 1-undecene	22	0.0066
daf-1(m40)	+ 1-undecene	21	- 1-undecene	25	0.9948
daf-1(m40)	+ 1-undecene	21	- 1-undecene	23	0.2990
daf-7(e1372)	+ 1-undecene	21	- 1-undecene	21	0.4488
daf-7(e1372)	+ 1-undecene	21	- 1-undecene	23	0.7628
N2	+ pyrrole	24	- pyrrole	19	<0.0001
N2	+ pyrrole	22	- pyrrole	22	0.0046
xbp-1(zc12)	+ pyrrole	17	- pyrrole	17	0.0071
xbp-1(zc12)	+ pyrrole	19	- pyrrole	17	<0.0001
xbp-1(zc12)	+ pyrrole	22	- pyrrole	17	<0.0001

Supplementary Table 2. List of *C. elegans* strains used.

***Caenorhabditis elegans* strains used**		
Wild type	CGC	N2
zcIs4 [hsp-4::GFP] V	CGC	SJ4005
ire-1(zc14) II*; zcIs4 [hsp-4::GFP]* V	CGC	SJ30
zIs356 [daf-16p::daf-16a/b::GFP + rol-6(su1006)] IV	CGC	TJ356
rmIs132 [unc-54p::Q35::YFP]	CGC	AM140
dvIn70 [*hsp-16-2p::GFP*; *rol-6*]	CGC	CL2070
daf-11(m47) V	CGC	DR47
daf-1 (m40) IV	CGC	DR40
daf-7 (e1372) III	CGC	CB1372
rmIs110 [F25B3.3p::Q40::YFP]	Imanikia *et al.*, 2019	AGD1397
uthIs393 [vha-6p::Q40::YFP+rol-6(su1006)]	Imanikia *et al.*, 2019	AGD1395
rmsIs9 [*daf-7p::xbp-1s::unc-54* 3'UTR, *myo-3p::mKate*]; *zcIs4 [hsp-4p::GFP]* V	Özbey *et al.*, 2020	RCT206
rmsIs8 [xbp-1p::xbp-1::GFP]	Özbey *et al.*, 2020	RCT21
rmsIs7 [*tdc-1p::mKate2::let-858* 3'UTR, *cc::GFP*]; *rmsIs8* [*xbp-1p::xbp-1::GFP*]	Özbey *et al.*, 2020	RCT192
xbp-1 (zc12) III	Taylor & Dillin, 2013	AGD1049
xbp-1 (zc12) III; *zcIs4 [hsp-4::GFP]* V	Taylor & Dillin, 2013	AGD972
unc-13 (e450) I; *zcIs4 [hsp-4::GFP]* V	Taylor & Dillin, 2013	AGD1137

drcSI7 [*daf-7p::Venus*]	Tullet Lab, U. of Kent	JMT50
daf-1 (m40) IV*; zcls4 [hsp-4::GFP]* V*; ftEx205[tdc-1p::daf-1:gfp + odr-1:dsRED]*	This study	RCT378
pmk-1 (k25) IV*; zcls4 [hsp-4::GFP]* V	This study	RCT379
kgb-1 (um3) IV*; zcls4 [hsp-4::GFP]* V	This study	RCT380
lim-4 (ky403); zcls4 [hsp-4::GFP] V	This study	RCT381
oyIs84 [gpa-4p::TU#813 + gcy-27p::TU#814 + gcy-27p::GFP + unc-122p::DsRed]; zcls4 [hsp-4::GFP] V	This study	RCT237
daf-7 (e1372) III*; rmls132 [unc-54p::Q35::YFP]*	This study	RCT382
zip-2 (ok3730) III*; zcls4 [hsp-4::GFP]* V	This study	RCT369
unc-31 (e928) IV*; zcls4 [hsp-4::GFP]* V	This study	RCT370
tdc-1 (n3419) II*; zcls4 [hsp-4::GFP]* V	This study	RCT66
tph-1 (mg280) II; *zcls4 [hsp-4::GFP]* V	This study	RCT371
cat-2 (*e1112*) II; *zcls4 [hsp-4::GFP]* V	This study	RCT372
unc-25 (e156) III; *zcls4 [hsp-4::GFP]* V	This study	RCT373
eat-4 (ky5) III; *zcls4 [hsp-4::GFP]* V	This study	RCT374
nsIs180 [hlh-17p::recCaspase-3, unc-122p::GFP]; zcls4 [hsp-4::GFP] V	This study	RCT375
cho-1 (tm373) IV; *zcls4 [hsp-4::GFP]* V	This study	RCT189
alh-11 (lj118) III; *zcls4 [hsp-4::GFP]* V	This study	RCT376
daf-1 (m40) IV; *zcls4 [hsp-4::GFP]* V	This study	RCT67
daf-7 (e1372) III; *zcls4 [hsp-4::GFP]* V	This study	RCT68
odr-3 (rms31) V*; zcls4 [hsp-4::GFP]* V	This study	RCT377

Supplementary Table 3. List of qPCR primers used in this work.

	**SOURCE**	**IDENTIFIER**
**qPCR Primers**		
GTTCCCGTGTTCATCACTCAT	Sigma	*pmp-3* F
ACACCGTCGAGAAGCTGTAGA	Sigma	*pmp-3* R
CTGCTGGACAGGAAGATTACG	Sigma	*cdc-42* F
CTCGGACATTCTCGAATGAAG	Sigma	*cdc-42* R
GTCGCTTCAAATCAGTTCAGC	Sigma	*Y45F10D.4* F
GTTCTTGTCAAGTGATCCGACA	Sigma	*Y45F10D.4* R
CGTGCCTTTGAATCAGCAGTG	Sigma	*xbp-1*_s F
CGAGGTGTCCATCTTCTTGTT	Sigma	*xbp-1*_s R
CAGATGAAAACTCAAATCGCC	Sigma	*hsp-4* F
GGTTGCTTCCGAGCCACTCAA	Sigma	*hsp-4* R
CTTTATTCGCTCCCTAACCGT	Sigma	*Y41C4A.11* F
CTGCTCCATTCTCCACTCGTT	Sigma	*Y41C4A.11* R
TTCTACACCGCCAAAGATCC	Sigma	*daf-7* F
CTGTGAGTGTGGCCTGAAGA	Sigma	*daf-7* R

Supplementary Table 4. List of CRISPR primers and oligonucleotides used in this work.

	SOURCE	IDENTIFIER
**CRISPR Oligos**		
GCTACCATAGGCACCACGAG	IDT	*dpy-10* cRNA
AAAATTCGGAAGGTAACGCG; C<br>TGCATTTACCGTTGGAAAA	IDT	*odr-3* cRNAs
CACTTGAACTTCAATACGGCAAGATGAG<br>AATGACTGGAAACCGTACCGCATGCGG<br>TGCCTATGGTAGCGGAGCTTCACATGGC<br>TTCAGACCAACAGCCTAT	IDT	*dpy-10* Repair Template
caattactcatagatcattgtttttttagATATGGGCTCA<br>TGCCAGAGCAATGAAAATTCGGAAGGTA<br>ACAAATGGACAAAAAGAAGCAGAAAAGG<br>CAATAGTTATGAAAGTACAGGAAAATGGAGAAG<br>AAGGAGAAGCACTGACAGAAGAAGTTTCGAAA<br>GCAATTCAATCGTTATGGGCAGATCCTGGCGTGAAGAA	IDT	*odr-3* Repair Template
