# Supplementary material for: Olfactory chemosensation extends lifespan through TGF-β signaling and UPR activation
Source: Nat Aging. 2023 Jul 27;3(8):938–47. doi: 10.1038/s43587-023-00467-1 (PMC10432268; doi:10.1038/s43587-023-00467-1)
Supplement: Supplementary file 2 — Reporting Summary [file 43587_2023_467_MOESM2_ESM.pdf]

nature portfolio

Double-blind peer review submissions: write DBPR and your manuscript number here instead of author names.

Corresponding author(s): Evandro A. De-Souza, Maximillian A. Thompson, Rebecca C. Taylor

Last updated by author(s): 2023-06-20

Reporting Summary

Nature Portfolio wishes to improve the reproducibility of the work that we publish. This form provides structure for consistency and transparency in reporting. For further information on Nature Portfolio policies, see our Editorial Policies and the Editorial Policy Checklist.

Statistics

For all statistical analyses, confirm that the following items are present in the figure legend, table legend, main text, or Methods section.

n/a	Confirmed	
[ ]	[x]	The exact sample size (n) for each experimental group/condition, given as a discrete number and unit of measurement
[ ]	[x]	A statement on whether measurements were taken from distinct samples or whether the same sample was measured repeatedly
[ ]	[x]	The statistical test(s) used AND whether they are one- or two-sided<br>*Only common tests should be described solely by name; describe more complex techniques in the Methods section.*
x [ ]	[x]	A description of all covariates tested
[ ]	[x]	A description of any assumptions or corrections, such as tests of normality and adjustment for multiple comparisons
[ ]	[x]	A full description of the statistical parameters including central tendency (e.g. means) or other basic estimates (e.g. regression coefficient) AND variation (e.g. standard deviation) or associated estimates of uncertainty (e.g. confidence intervals)
[ ]	[x]	For null hypothesis testing, the test statistic (e.g. F, t, r) with confidence intervals, effect sizes, degrees of freedom and P value noted<br>*Give P values as exact values whenever suitable.*
[x]	[ ]	For Bayesian analysis, information on the choice of priors and Markov chain Monte Carlo settings
[x]	[ ]	For hierarchical and complex designs, identification of the appropriate level for tests and full reporting of outcomes
[x]	[ ]	Estimates of effect sizes (e.g. Cohen's d, Pearson's r), indicating how they were calculated

Our web collection on statistics for biologists contains articles on many of the points above.

Software and code

Policy information about availability of computer code

Data collection	Leica LAS X (version 5.1.0)
Data analysis	Fiji - ImageJ distro(version 1.53e) GraphPad Prism (version 8.4.2)

For manuscripts utilizing custom algorithms or software that are central to the research but not yet described in published literature, software must be made available to editors and reviewers. We strongly encourage code deposition in a community repository (e.g. GitHub). See the Nature Portfolio guidelines for submitting code & software for further information.

Data

Policy information about availability of data

All manuscripts must include a data availability statement. This statement should provide the following information, where applicable:

- Accession codes, unique identifiers, or web links for publicly available datasets
- A description of any restrictions on data availability
- For clinical datasets or third party data, please ensure that the statement adheres to our policy

Data is available upon request from the lead author - Rebecca Taylor. All data supporting the findings of this study are available within the paper and in the supplementary files

Human research participants

Policy information about studies involving human research participants and Sex and Gender in Research.

Reporting on sex and gender	*Use the terms sex (biological attribute) and gender (shaped by social and cultural circumstances) carefully in order to avoid confusing both terms. Indicate if findings apply to only one sex or gender; describe whether sex and gender were considered in study design whether sex and/or gender was determined based on self-reporting or assigned and methods used. Provide in the source data disaggregated sex and gender data where this information has been collected, and consent has been obtained for sharing of individual-level data; provide overall numbers in this Reporting Summary. Please state if this information has not been collected. Report sex- and gender-based analyses where performed, justify reasons for lack of sex- and gender-based analysis.* N/A
Population characteristics	*Describe the covariate-relevant population characteristics of the human research participants (e.g. age, genotypic information, past and current diagnosis and treatment categories). If you filled out the behavioural & social sciences study design questions and have nothing to add here, write "See above."* N/A
Recruitment	*Describe how participants were recruited. Outline any potential self-selection bias or other biases that may be present and how these are likely to impact results.* N/A
Ethics oversight	*Identify the organization(s) that approved the study protocol.* N/A

Note that full information on the approval of the study protocol must also be provided in the manuscript.

Field-specific reporting

Please select the one below that is the best fit for your research. If you are not sure, read the appropriate sections before making your selection.

☒ Life sciences ☐ Behavioural & social sciences ☐ Ecological, evolutionary & environmental sciences

For a reference copy of the document with all sections, see nature.com/documents/nr-reporting-summary-flat.pdf

Life sciences study design

All studies must disclose on these points even when the disclosure is negative.

Sample size	Sample size was determined based upon norms in the field, most analysis was via microscopy (approx 50 animals/condition). qPCR etc was determined by field norms, usually going beyond these samples sizes where RNA was available to do so. No statistical methods were used to pre-determine sample sizes but our sample sizes are similar to those reported in previous publication from our group (Imanikia et al., 2019)
Data exclusions	Outliers as determined by Grubbs test for outliers were excluded from qPCR data throughout. No animals were excluded from the analysis. However, some data from the RNA analysis that did not meet the pre-determined quality control standards (260/280 ratio) were excluded.
Replication	Each experiment shown was repeated for N=3 and representative trials are shown rather than pooled data. Exceptions to this are qPCR data where multiple biological replicates were used. Unless specified differently in the figure legend, a minimum of three individual experiments were conducted for each assay. All attempts of replication yielded similar results.
Randomization	Animals were randomly selected based upon developmental stage and not screened in any way prior to analysis. Importantly we did not look at the animals under fluorescence before selecting animals for microscopy for example. Animals were randomly selected based upon developmental stage and not screened in any way prior to analysis.
Blinding	Lifespans were performed blinded, due to manpower constraints microscopy was not blinded but worms were selected randomly for analysis.

Reporting for specific materials, systems and methods

We require information from authors about some types of materials, experimental systems and methods used in many studies. Here, indicate whether each material, system or method listed is relevant to your study. If you are not sure if a list item applies to your research, read the appropriate section before selecting a response.

Materials & experimental systems

n/a	Involved in the study	
☒	☐	Antibodies
☒	☐	Eukaryotic cell lines
☒	☐	Palaeontology and archaeology
☐	☒	Animals and other organisms
☒	☐	Clinical data
☒	☐	Dual use research of concern

Methods

n/a	Involved in the study	
☒	☐	ChIP-seq
☒	☐	Flow cytometry
☒	☐	MRI-based neuroimaging

March 2021

Animals and other research organisms

Policy information about studies involving animals; ARRIVE guidelines recommended for reporting animal research, and Sex and Gender in Research

Laboratory animals	Caenorhabiditis Elegans
Wild animals	*Provide details on animals observed in or captured in the field; report species and age where possible. Describe how animals were caught and transported and what happened to captive animals after the study (if killed, explain why and describe method; if released, say where and when) OR state that the study did not involve wild animals.* This study did not use wild animals
Reporting on sex	Hermaphrodite animals analyzed throughout.
Field-collected samples	*For laboratory work with field-collected samples, describe all relevant parameters such as housing, maintenance, temperature, photoperiod and end-of-experiment protocol OR state that the study did not involve samples collected from the field.* No field-collected samples were used in this study
Ethics oversight	*Identify the organization(s) that approved or provided guidance on the study protocol, OR state that no ethical approval or guidance was required and explain why not.* No ethical approval is required for studies with Caenorhabditis elegans

Note that full information on the approval of the study protocol must also be provided in the manuscript.

Laboratory animals:

Caenorhabditis elegans hermaphrodites at day 1 of adulthood were used in all experiments, unless otherwise specified (in the figure lends).

The C. elegans strains used were:

Wild type N2

zcls4 [hsp-4::GFP] V SJ4005

ire-1(zc14) II; zcls4 [hsp-4::GFP] V SJ30

zls356 [daf-16p::daf-16a/b::GFP + rol-6(su1006)] IV TJ356

rmls132 [unc-54p::Q35::YFP] AM140

dvln70 [hsp-16-2p::GFP; rol-6] CL2070

daf-11(m47) V DR47

daf-1 (m40) IV DR40

daf-7 (e1372) III CB1372

rmls110 [F25B3.3p::Q40::YFP] AGD1397

uthls393 [vha-6p::Q40::YFP+rol-6(su1006)] AGD1395

rmsls9 [daf-7p::xbp-1s::unc-54 3'UTR, myo-3p::mKate]; zcls4 [hsp-4p::GFP] V RCT206

rmsls8 [xbp-1p::xbp-1::GFP] RCT21

rmsls7 [tdc-1p::mKate2::let-858 3'UTR, cc::GFP]; rmsls8 [xbp-1p::xbp-1::GFP] RCT192

xbp-1 (zc12) III AGD1049

xbp-1 (zc12) III; zcls4 [hsp-4::GFP] V AGD972

unc-13 (e450) I; zcls4 [hsp-4::GFP] V AGD1137

drcSI7 [daf-7p::Venus] JMT50

daf-1 (m40) IV; zcls4 [hsp-4::GFP] V; ftEx205[tdc-1p::daf-1:gfp + odr-1:dsRED] RCT378

pmk-1 (k25) IV; zcls4 [hsp-4::GFP] V RCT379

kgb-1 (um3) IV; zcls4 [hsp-4::GFP] V RCT380

lim-4 (ky403); zcls4 [hsp-4::GFP] V RCT381

oyls84 [gpa-4p::TU#813 + gcy-27p::TU#814 + gcy-27p::GFP + unc-122p::DsRed]; zcls4 [hsp-4::GFP] V RCT237

daf-7 (e1372) III; rmls132 [unc-54p::Q35::YFP] RCT382

zip-2 (ok3730) III; zcls4 [hsp-4::GFP] V RCT369

unc-31 (e928) IV; zcls4 [hsp-4::GFP] V RCT370

tdc-1 (n3419) II; zcls4 [hsp-4::GFP] V RCT66

tph-1 (mg280) II; zcls4 [hsp-4::GFP] V RCT371

cat-2 (e1112) II; zcls4 [hsp-4::GFP] V RCT372
